# Nature, extent and ecological implications of night‐time light from road vehicles

**DOI:** 10.1111/1365-2664.13157

**Published:** 2018-04-25

**Authors:** Kevin J. Gaston, Lauren A. Holt

**Affiliations:** ^1^ Environment & Sustainability Institute University of Exeter Cornwall UK; ^2^ Wissenschaftskolleg zu Berlin, Institute for Advanced Study Berlin Germany

**Keywords:** artificial light, light cycles, light pollution, night‐time, skyglow, spectra, urban ecology, vehicles

## Abstract

1. The erosion of night‐time by the introduction of artificial lighting constitutes a profound pressure on the natural environment. It has altered what had for millennia been reliable signals from natural light cycles used for regulating a host of biological processes, with impacts ranging from changes in gene expression to ecosystem processes.
2. Studies of these impacts have focused almost exclusively on those resulting from stationary sources of light emissions, and particularly streetlights. However, mobile sources, especially road vehicle headlights, contribute substantial additional emissions.
3. The ecological impacts of light emissions from vehicle headlights are likely to be especially high because these are (1) focused so as to light roadsides at higher intensities than commonly experienced from other sources, and well above activation thresholds for many biological processes; (2) projected largely in a horizontal plane and thus can carry over long distances; (3) introduced into much larger areas of the landscape than experience street lighting; (4) typically broad “white” spectrum, which substantially overlaps the action spectra of many biological processes and (5) often experienced at roadsides as series of pulses of light (produced by passage of vehicles), a dynamic known to have major biological impacts.
4. The ecological impacts of road vehicle headlights will markedly increase with projected global growth in numbers of vehicles and the road network, increasing the local severity of emissions (because vehicle numbers are increasing faster than growth in the road network) and introducing emissions into areas from which they were previously absent. The effects will be further exacerbated by technological developments that are increasing the intensity of headlight emissions and the amounts of blue light in emission spectra.
5. *Synthesis and applications*. Emissions from vehicle headlights need to be considered as a major, and growing, source of ecological impacts of artificial night‐time lighting. It will be a significant challenge to minimise these impacts whilst balancing drivers' needs at night and avoiding risk and discomfort for other road users. Nonetheless, there is potential to identify solutions to these conflicts, both through the design of headlights and that of roads.

## Abstract 2

Emissions from vehicle headlights need to be considered as a major, and growing, source of ecological impacts of artificial night‐time lighting. It will be a significant challenge to minimise these impacts whilst balancing drivers' needs at night and avoiding risk and discomfort for other road users. Nonetheless, there is potential to identify solutions to these conflicts, both through the design of headlights and that of roads.

## 1 INTRODUCTION

Artificial lighting of the night‐time has brought enormous benefits to humankind, and has shaped societies in dramatic ways. Indeed, over the last hundred years or so, the introduction of electric street lighting in particular into villages, towns, and cities, has come to epitomise development and modernity. There has been rapid, and ongoing, expansion of the extent of the global area that is now directly artificially lit, including into those parts of landscapes, the protected areas, that are meant to be best shielded from anthropogenic influences (Davies, Duffy, Bennie, & Gaston, 2016; Gaston, Duffy, & Bennie, 2015). Skyglow, caused predominantly by upwardly emitted artificial light being scattered in the atmosphere, and which may reach 10s to 100s of kilometres beyond the limits of urban settlements (Biggs, Fouché, Bilki, & Zadnik, 2012; Luginbuhl, Boley, & Davis, 2014), is now estimated to be experienced by *c*. 23% of the global land area (Falchi et al., 2016).

This erosion of the night‐time has constituted a profound pressure on the natural environment. It has disrupted the natural daily and seasonal light cycles experienced by organisms in ways that have no natural analogues (Gaston, Visser, & Hölker, 2015). This has altered what had for millennia been reliable signals used for regulating a host of biological processes. An extraordinary array of impacts have now been documented, including on gene expression, the physiology and behaviour of organisms, the abundance and distribution of species, their ecological interactions, the composition of communities, and ecosystem processes and services (for a range of recent examples see Altermatt & Ebert, 2016; Davies et al., 2017; ffrench‐Constant et al., 2016; Raap, Pinxten, & Eens, 2016; Robert, Lesku, Partecke, & Chambers, 2015; Sanders et al., 2015; Thums et al., 2016; Wakefield, Stone, Jones, & Harris, 2015). Moreover, these effects have been found across a wide diversity of species, including microbes, plants, molluscs, arachnids, insects, fish, amphibians, reptiles, birds and mammals (Bennie, Davies, Cruse, & Gaston, 2016; Gaston, Bennie, Davies, & Hopkins, 2013). In consequence, potential mitigation measures (e.g. dimming of emissions, partial night‐time lighting, shielding light sources, modifying emission spectra) have been much discussed, and there are growing numbers of examples of their implementation (Azam et al., 2015; Falchi, Cinzano, Elvidge, Keith, & Haim, 2011; Gaston, Davies, Bennie, & Hopkins, 2012).

Research into the ecological and evolutionary impacts of artificial night‐time lighting, and how these can best be minimised, has focused almost exclusively on emissions from streetlights. Studies have variously (1) conducted field observations to determine the impacts of street lighting emissions (e.g. Davies, Bennie, & Gaston, 2012; Kempenaers, Borgström, Löes, Schlicht, & Valcu, 2010; Mathews et al., 2015); (2) introduced streetlights into previously unlit areas in experiments to determine their impacts (e.g. de Jong et al., 2015; Hölker et al., 2015; Spoelstra et al., 2015) and (3) simulated the emissions from streetlights in either laboratory or field experiments to determine their effects (e.g. Bennie, Davies, Cruse, Bell, & Gaston, 2018; Bennie, Davies, Cruse, Inger, & Gaston, 2015; Davies et al., 2017; Sanders et al., 2015). However, whilst streetlights are a major source of artificial night‐time lighting, they are far from the only one. A few studies have examined ecological impacts of some other stationary sources (e.g. communication towers, lighthouses; Jones & Francis, 2003; Longcore et al., 2012), but the ecological impacts of mobile sources of lighting have remained virtually ignored.

The predominant mobile source of artificial night‐time light is the emissions from vehicle, and particularly road vehicle, headlights. The ecological impacts that might arise from these have received almost no attention, or only passing reference, either within the literature on impacts of artificial night‐time lighting (e.g. see reviews by Longcore & Rich, 2004; Gaston et al., 2013; Gaston, Duffy, Gaston, Bennie, & Davies, 2014), or on the ecological impacts of roads (e.g. see reviews by Coffin, 2007; Spellerberg, 1998; Trombulak & Frissell, 2000; van der Ree, Smith, & Grilo, 2015). Where they have been considered, the focus has been on the dazzling of vertebrates and the resultant potential for these causing collisions with vehicles (e.g. Outen, 2002). Notwithstanding, there are good reasons to predict that headlight emissions have profound ecological impacts, both because of their general contribution to artificial night‐time lighting, and because of the particular challenges posed by the high intensity of their emissions and the pulse‐like nature of illuminance caused by passing vehicles.

In this paper, we review the nature, extent and ecological implications of artificial light from vehicle headlights. We do so by exploring in turn each of four key issues that shape the ecological impacts of artificial night‐time lighting, namely light intensity, spectrum, spatial extent and temporal pattern. Essentially, we work from the level of individual vehicles to that of the landscape, and explore the ways in which recent and potential developments in vehicle ownership and technology may influence these effects.

## 2 INTENSITY

### 2.1 Background

**Figure 1:**
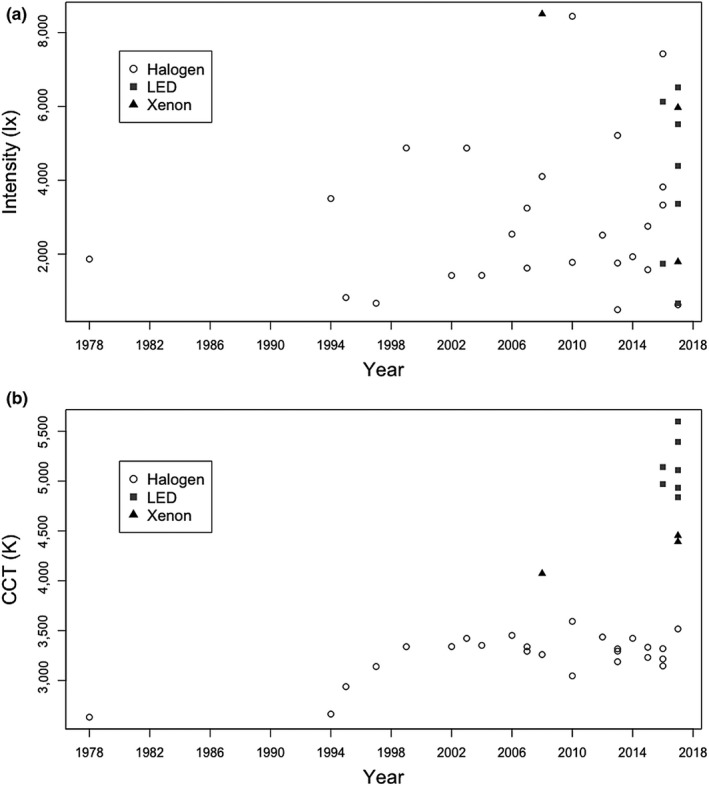
Variation in (a) intensity and (b) correlated colour temperature (CCT) of emissions measured from headlights on full beam for different makes and models of cars, of a variety of ages (year) (*n* = 35). CCT is the absolute temperature of a blackbody whose chromaticity most nearly resembles that of the light source, and is frequently used to describe the aesthetic appearance of white light, from “warm” orange to “cool” blue light. Symbols represent light type. Data were collected using a UPRtek MK350N PLUS spectrometer, held in a cushioned frame that was placed in a standardised way directly on car headlights and surrounded by blackout fabric that eliminated external ambient light in the visible spectrum. These figures represent forward emissions and not the peak emissions achieved by the angling and reflection of the light. Some of the variation in figures is likely to be due to the shape and configuration of headlight assemblies

**Figure 2:**
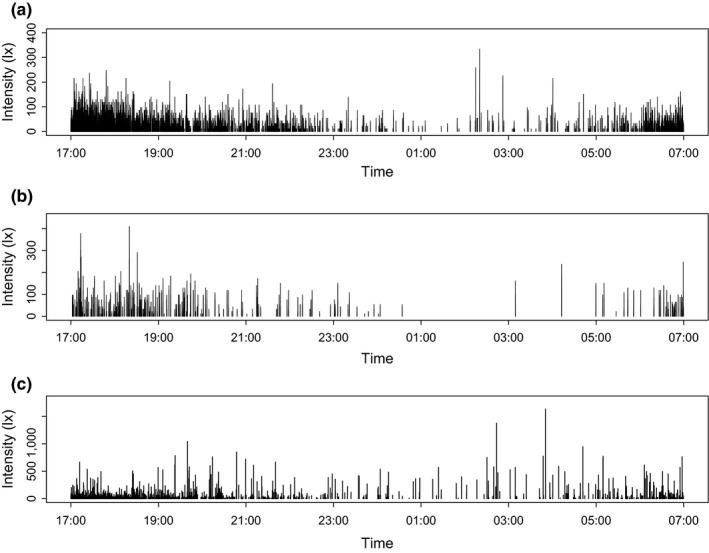
Light intensity over example night‐time 18 hr periods at three road sides in Cornwall, U.K. (a) Link road to Treluswell: 50°10′23.0″N 5°07′46.0″W (b) Road to Laddock: 50°17′34.1″N 4°58′09.5″W (c) Link to A30 (on corner): 50°17′27.8″N 5°02′34.1″W. Measurements were made on 19 December 2016 (under overcast conditions with light rain), using Onset Hobo^®^ UA‐002‐64 pendant light recorders, placed at 55 cm above‐ground level, secured to wooden posts and pointed in the direction of oncoming traffic. Posts were placed 3 m from the midline of the oncoming traffic lane

Typical intensities of light emissions measured directly from headlights are around 2,000‐8,000 lx for newer cars, but can be higher (Figure 1); lux (lx) is a measure of luminous flux per unit area based on human photopic vision, and so does not necessarily capture the relative effects of light influencing biological processes with different spectral responses, but its use ensures a direct link to illuminance as commonly measured in the environment and employed in the design and mitigation of artificial lighting systems. This level of luminance is broadly comparable to that from emissions measured directly from streetlights, but vehicle headlights have a much more focused beam, which travels further at higher intensities. Therefore, whilst the downward directed emissions from streetlights tend to result in ground‐level illuminance of around 10–20 lx directly below the source, which usually declines to <1 lx a few metres away, those from vehicles reach much higher levels over much greater distances, both horizontally and vertically. For example, emissions for a family car that approached 10,000 lx at source remained at 25 lx at 50 m distance, and exceeded 1 lx at 100 m; moonlight is *c*. 0.1 lx (for a full moon) (Bennie et al., 2016). As a result, roadside vegetation and the surrounding area is frequently illuminated at night by emissions at levels of the order of 300 lx, and, depending on the angle to the oncoming traffic and the likelihood of vehicles using full beam (which will tend to be higher on rural roads, given lower levels of traffic), this may on occasion approach levels of around 1,000 lx or more, equivalent to daylight on a heavily overcast day (Figure 2).

The artificial night‐time lighting emitted by streetlights has been shown regularly to exceed the thresholds, which are often low (<1 lx; Gaston et al., 2013, 2014), that trigger a wide variety of biological effects (e.g. physiological, behavioural, and other responses); this includes attraction and repulsion behaviours of animals, which may or may not influence risks of vehicle collision. Nonetheless, dose‐response relations—how effects change with increasing intensity of emissions—are poorly understood for most of these effects, and research establishing them is regarded as a high priority (Gaston et al., 2015). The yet greater levels of illuminance at distance from vehicle headlights mean that the upper intensity levels that require exploration will need to be substantially higher than those from streetlights, and than the intensities which have been used in empirical studies thus far (e.g. Bennie et al., 2015; Davies et al., 2017; de Jong et al., 2016; Sanders et al., 2015).

### 2.2 Developments

The history of vehicle headlights has largely been one in which the intensity of emissions has progressively increased with technological improvements and innovations. The maximum intensity allowed along the axis of a single headlamp on full‐beam (or high‐beam) is presently 112,500 cd in Europe (under ECE Regulation 48) and Japan (under Japanese Safety Regulation Article 32), and 75,000 cd in the U.S. (under Federal Vehicle Motor Standard 108; Rumar, 2000); *E *= *I*/(*d*)^2^, where *E* is intensity of emissions in lux (lx), *I* the intensity in candelas (cd), and *d* is distance in metres. In general, regulations have tended to increase to keep track with vehicle headlight strength, whilst keeping below a level that creates too much glare for drivers of oncoming vehicles.

Recent technological developments are seeing the replacement of halogen bulbs with high intensity discharge (HID) xenon, light‐emitting diode (LED) and, in as yet a very limited way, laser light sources, all of which can reach greater visible outputs. Laser headlights can produce an exceptionally bright white light that is significantly more intense than conventional light sources. Yet, in tacit acknowledgement of the potential for unprecedented levels of vehicular light pollution, laser headlights are not currently authorised in urban areas. In time the current “higher‐end” technologies of LED and laser will become more affordable, and will be incorporated into low and medium cost vehicles (LED bulbs are already widely available for retrofitting into vehicle headlight assemblies).

Unless road vehicle technology changes in such a way as to make headlights redundant (see below), there is little evidence that emissions will not continue to increase with further innovations in headlight technology. This said, one potential brake on increasing intensity of emissions from new types of headlights may arise from concerns that these exacerbate effects of glare from oncoming vehicles, particularly for older drivers and in ageing populations. HID lights are especially problematic in this regard. The effect is worse for older drivers due to increased intraocular light scattering, glare sensitivity, and photostress recovery time (Mainster & Timberlake, 2003).

## 3 SPECTRUM

### 3.1 Background

**Figure 3:**
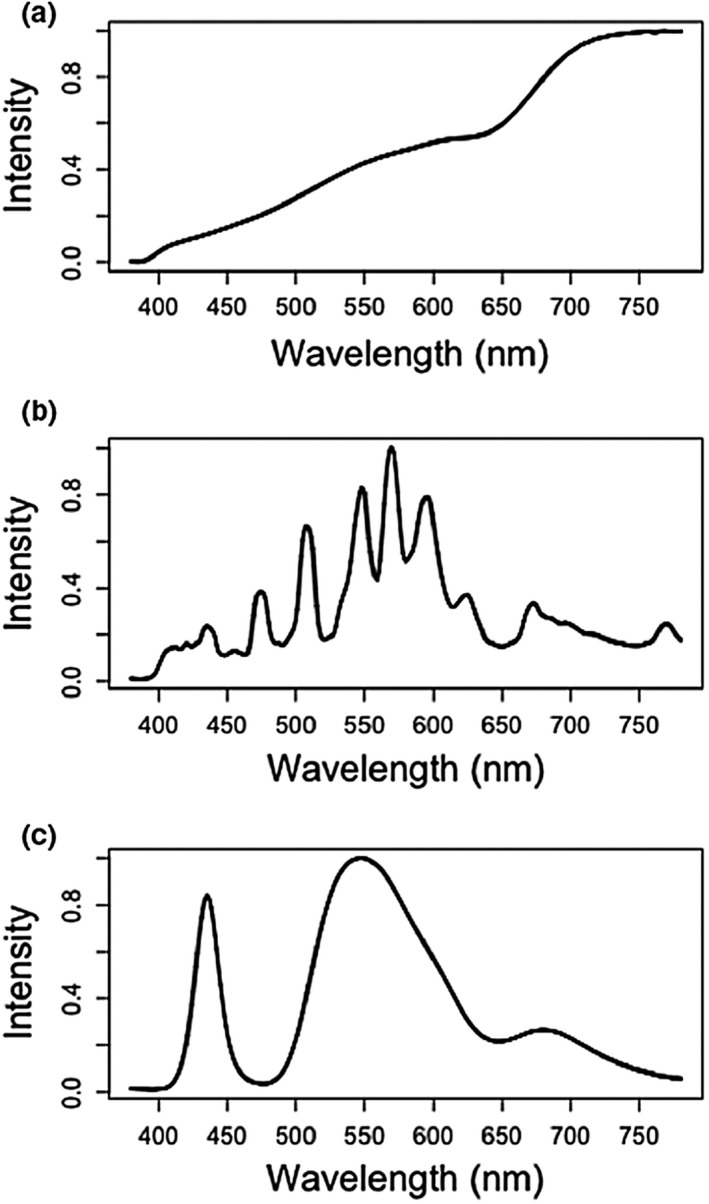
Measured spectral irradiances (relative intensity) of three contrasting headlight types: (a) halogen; (b) high intensity discharge xenon; and (c) “white” light‐emitting diode. Data were collected using a UPRtek MK350N PLUS spectrometer, held in a cushioned frame that was placed directly on car headlights and surrounded by blackout fabric

Streetlight emissions are very different in their spectra from sunlight, moonlight or starlight (Gaston et al., 2014). Some types emit over very narrow bandwidths (e.g. low‐pressure sodium lighting), others do so over a wide range of wavelengths (e.g. high‐pressure sodium lighting, “white” LED lighting; Elvidge, Keith, Tuttle, & Baugh, 2010). Current vehicle headlight types tend to be of the latter form (Figure 3). Of those in present use, halogen lights (a type of incandescent lamp) are the oldest and commonest, and have a broader spectrum with greater emissions towards the longer visible wavelengths (Figure 3a). Xenon lights have peaks over a range of shorter to intermediate visible wavelengths (Figure 3b). LED lights typically have peaks in the blue and green (Figure 3c). Laser headlights are not currently widely commercially available and the details of the spectra remain unclear, but they provide focused, high‐contrast white light intended to mimic sunlight, and are adapted from blue‐laser diode technology (Wierer, Tsao, & Sizov, 2013).

Key to the ecological impact of artificial night‐time lighting is the interaction between the spectral composition of that lighting and the action spectra of biological processes (Aubé, Roby, & Kocifaj, 2013; Davies, Bennie, Inger, Hempel de Ibarra, & Gaston, 2013; Solano Lamphar & Kocifaj, 2013). These action spectra vary a great deal both between different processes (e.g. Ahmad et al., 2002; Aubé et al., 2013; Butler, Hendricks, & Siegelman, 1964) and between different kinds of organisms for a given process (e.g. photosynthesis: Clark & Lister, 1975; Inada, 1976; vision: Davies et al., 2013; Solano Lamphar & Kocifaj, 2013). Research is being conducted to determine the impacts of artificial night‐time lighting on systems with a range of different action spectra (e.g. Aubé et al., 2013; Davies et al., 2013; Solano Lamphar & Kocifaj, 2013). White light is typical for car headlights for superior illumination at night, to avoid causing unnecessary fatigue, and to avoid inhibiting driver's colour vision. However, it is widely held that broad “white” lighting is environmentally especially problematic because of the greater likelihood of substantial emissions in key parts of the action spectra of many biological processes. Concerns have particularly been raised around emissions in the blue part of the spectrum, which have marked influences on melatonin levels and circadian rhythms of many species (Bayarri, Madrid, & Sánchez‐Vázquez, 2002; Lockley, Brainard, & Czeisler, 2003), and are more attractive to some organisms (e.g. Cowan & Gries, 2009; Evans, Akashi, Altman, & Manville, 2007; Somers‐Yeates, Hodgson, McGregor, Spalding, & ffrench‐Constant, 2013) whilst being more repellent to others (e.g. Downs et al., 2003; Widder, Robison, Reisenbichler, & Haddock, 2005). Car headlights have progressively increased in their correlated colour temperature (CCT) values (frequently used to describe the aesthetic appearance of white light, increasing in value from “warm” orange to “cool” blue light). Newer headlight types, particularly xenon and LED, have substantial emissions at blue wavelengths (intended to help drivers pick out objects and ease eye fatigue; Mainster & Timberlake, 2003); several new LED headlights are close to the international regulated limit of 6,000 K (Figure 1).

### 3.2 Developments

It seems likely that there will be increasing use of headlight technologies with greater emissions particularly in the biologically significant blue part of the spectrum. A similar shift has been seen in streetlight technology, and has led to much public discussion over the implications for human health and wellbeing, for aesthetics, and for wider environmental impacts. In particular, there has been public opposition in some areas to the use of LED street lighting with higher CCT values. It would seem sensible to bring the desirability of developments in headlight technology into these same debates. The breadth of concerns here is similarly wide, embracing not just potential environmental impacts, but those on the behaviour and wellbeing of oncoming drivers and pedestrians, on occupants of roadside properties, and on the night‐time aesthetics of roads.

## 4 SPATIAL EXTENT

### 4.1 Background

Vehicle headlights illuminate vastly greater areas of habitat than do streetlights, introducing artificial night‐time lighting into areas without streetlights or other static forms of lighting. For example, 238,000 ha of road verge alone exist in Britain, more than twice the area of natural and semi‐natural grassland in the wider countryside (Plantlife, 2013), and whilst outside of urban areas only a small proportion of this road verge is lit by streetlights, virtually all is lit at some time by vehicle headlights.

The global (paved and unpaved) road network is estimated to be more than 64 million km in length, with that of Brazil being 1.6 million km, China 4.1 million km, India 4.7 million km and the USA 6.6 million km (Central Intelligence Agency, 2013). In some parts of the world, this coverage is such that influences from roads are arguably the norm for areas rather than the exception. Of the coterminous United States, 20% of the total land area has been estimated to lie within 127 m of a road and 83% within 1,061 m (Riitters & Wickham, 2003).

**Figure 4:**
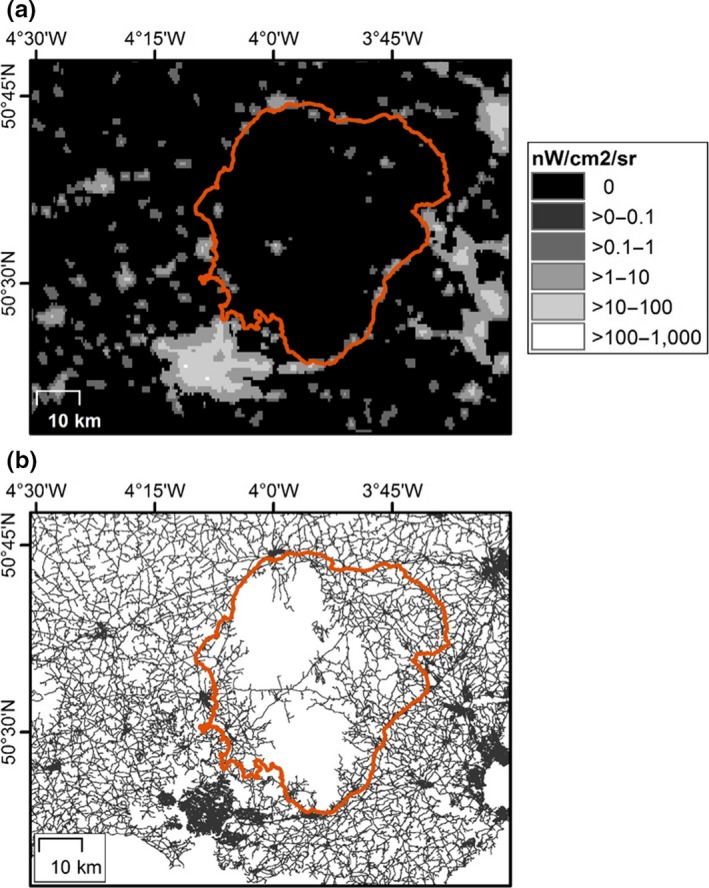
(a) Annual composite of night‐time lights from 2015 as recorded from the Visible Infrared Imaging Radiometer Suite (VIIRS) Day/Night Band (DNB) (Earth Observation Group & NOAA National Geophysical Data Center, 2017) and (b) Highways Agency road network (Ordnance Survey, 2016) for a region of Devon and East Cornwall including the rural area of Dartmoor National Park (delineated in red) and the city of Plymouth [Colour figure can be viewed at wileyonlinelibrary.com]

Headlight emissions are not captured well by the satellite imagery that is used widely to analyse spatial patterns of artificial night‐time lighting (Figure 4). This is both because the emissions occur predominantly in the horizontal plane, and because imagery is often processed to represent static/persistent lighting and remove ephemeral lighting (thereby avoiding contamination of images of artificial night‐time lighting with the location of fires etc.). In consequence, satellite imagery will tend markedly to underestimate the extent of artificial night‐time lighting. This is important, because such imagery has been used to determine the levels to which the night‐time environment has been eroded in different ecosystem types (Bennie, Duffy, Davies, Correa‐Cano, & Gaston, 2015; de Freitas, Bennie, Mantovani, & Gaston, 2017), across areas protected for conservation (Gaston et al., 2015), in areas with different species richness (Bennie, Duffy, Inger, & Gaston, 2014), and across the geographic ranges of different species (Duffy, Bennie, Durán, & Gaston, 2015). Almost invariably, concerns have been expressed as to the levels of light pollution being experienced, and the consequent changes in habitat suitability for organisms. However, in ignoring vehicle headlight emissions, these will tend to be substantial underestimates.

As well as causing direct illuminance, upwardly directed or reflected emissions from headlights will also contribute to skyglow, but these emissions are not presently incorporated into the prevailing models of this phenomenon, again underestimating its extent.

### 4.2 Developments

The spatial extent of influence of vehicle headlights is likely to be growing rapidly alongside that of the road network. Globally, this network increased by 35% in the decade 2000–2009, and it has been estimated that there will be a need for an additional 25 million km of paved roads by 2050 (Dulac, 2013). Inevitably, this will introduce emissions from vehicle headlights into substantial areas in which they have not previously occurred. Of particular concern is that much of this growth in roads is likely to be in regions with rapidly emerging economies (e.g. China, India), with non‐OECD countries expected to account for nearly 90% of the global growth in roadway infrastructure (Dulac, 2013). These regions include ones of high global importance for biodiversity and ecosystem services (Laurance et al., 2014).

Some change in the spatial extent of influence of headlights from individual vehicles may result from increased used of adaptive technologies that, for example, cause these lights to swivel to better illuminate bends in the road and that extend beams on straighter roads. However, at least in the immediate term, these effects seem likely to be small compared with the overall growth in length of roads and numbers of road vehicles. This may place a primacy on careful planning of where new roads are built so as, alongside other concerns, to limit the propagation of headlight emissions across landscapes, and to incorporate into their design landscape or habitat changes that block or reduce this spread of light. It seems likely that, cognisant of safety issues, landscape profiling and careful planting of appropriate vegetation (akin to sound barriers) could serve markedly to limit the propagation of emissions from headlights both along existing and new roads.

## 5 TEMPORAL DYNAMICS

### 5.1 Background

**Figure 5:**
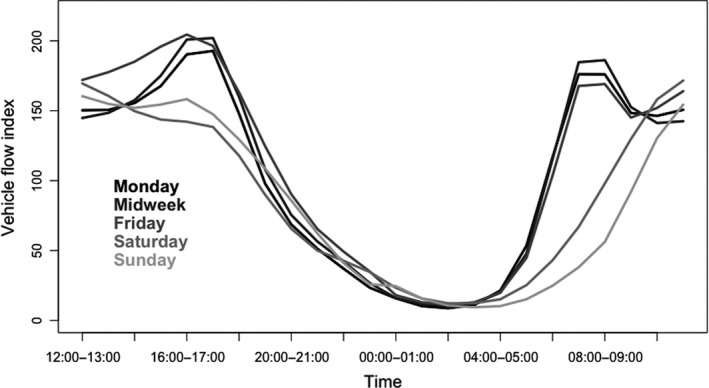
Traffic distribution by time of day on all roads, for cars, in Great Britain in 2015. This is scaled such that the average annual daily flow of 3,500 vehicles per day = index value of 100. Data from Department for Transport (2015)

Street lighting and other static forms of lighting give rise to night‐time‐long continuous or reasonably continuous periods of illumination. By contrast, at any one point along a roadside (and its surroundings) illuminance by emissions from headlights is typically pulsed due to the passage of vehicles (Figure 2). The form that the pulsing takes is dependent on the speed of vehicles, level of traffic and time of day. The greater the speed at which vehicles are moving, the briefer is the pulse of light received at a point along the roadside. For the majority of roads, the level of traffic varies markedly through the day (Figure 5), and in the UK, one of few regions for which data are accessible, 16%–48% of traffic is on the road outside of daylight hours depending on the time of year (Department for Transport, 2015). The volume of traffic, and thus the gaps between light pulses, also varies considerably throughout the night‐time, with relatively fewer journeys occurring in the early hours of the day (see Figure 5). The time of year may also have a significant effect; in winter organisms will experience more traffic‐related pulses due not only to a longer period of night‐time, but also because these dark or twilight hours are more likely to coincide with peak “rush‐hour” traffic. Furthermore, the level and pattern of traffic‐related light pulsing is likely to vary both regionally and globally, according to latitude (influencing seasonal variation in length of night‐time), level of economic development, and cultural conventions such as typical working and non‐working days of the week. This introduces a wholly unnatural regime of light exposure to organisms, unrelated to genuine seasonal or biological cues.

The vast majority of studies of the biological impacts of artificial night‐time lighting have focused on continuous lighting. However, pulsed lighting can have profound effects, at least as evidenced from laboratory studies. Table 1 provides examples of the findings of such studies, revealing that even brief single pulses of artificial night‐time lighting can be sufficient to induce a response. This table excludes the large number of studies that have used regular night‐time light pulses (commonly of 30 min or 1 hr duration, but sometimes much less) to produce phase response curves to understand the circadian rhythms of a variety of organisms (e.g. Daan & Pittendrigh, 1976; Flari & Lazaridou‐Dimitriadou, 1995; Ford & Cook, 1988; Gronfier, Wright, Kronauer, Jewett, & Czeisler, 2004; Kennedy & Hudson, 2016; Kumar & Singaravel, 2014). It also excludes phenomena such as the recovery times of night vision (“dark adaptation”) after exposure to artificial lighting (and associated “bleaching” of photopigments), which may in insects and vertebrates take 30 min or more (Martin, 2017; Post & Goldsmith, 1965), with profound consequences for resource acquisition and predator avoidance. Pulsed lighting has regularly been found to act as a repellent to organisms, with limited evidence for adaptive responses (e.g. Hamel, Brown, & Chipps, 2008; Linhart, 1984; Nemeth & Anderson, 1992; Patrick, Sheehan, & Sim, 1982; Sullivan et al., 2016), and to be less of an attractant than continuous lighting (Gehring, Kerlinger, & Manville, 2009). Areas experiencing pulsed lighting may thus as a consequence be avoided and may contribute to the fragmentation of habitats.

**Table 1:** Examples of the biological effects of pulsed night‐time lighting. Different studies use different measures of light intensity, many of which are not interchangeable

Species	Setting	Night‐time lighting	Effect	Source
Plants *Campanula carpatica*,* Coreopsis grandiflora*,* Petunia × hybrida*, and *Rudbeckia hirta*	Greenhouse	6 min pulse every 30 min for 4 hr using 600 W HPS lamp	≥80% of plants had macroscopic visible flower bud or inflorescence, whereas for all but one species controls remained vegetative	Blanchard and Runkle (2010)
Moths *Helicoverpa armigera* and *Mamestra brassicae*	Lab	0.5 s pulses of green light of 2.5 × 10^17^ photons.m^−2^.s^−1^ at 10 cm from source	Decreased activity in one species, no effect on other	Yabu, Miyashita, Uematsu, Wakukwa, and Arikawa (2014)
Mosquito *Anopheles gambiae*	Lab	6, 10 or 30 min pulses of 150, 300–870 lx	Suppression of biting activity	Sheppard et al. (2017)
Japanese horse‐mackerel *Trachuras japonicus*	Outdoor tank	Pulses of 3.0, 1.36, 0.62, and 0.15 cycles per second, at peak of 100 lx	General aversion to intermittent light or attraction and school confusion.	Koike and Matsuike (1987)
Senegal sole *Solea senegalensis*	Lab	1 hr pulse at 30 W	Decreased plasma melatonin	Bayarri et al. (2004)
Rat *Rattus norvegicus*	Lab	1 ms pulse at 2,000 mW/cm^2^	Pineal N‐actyltransferase & melatonin content reduced	Vollrath et al. (1989)
Rat *Rattus norvegicus*	Lab	5–60 min pulse every 2 hr at 200‐250 ft. cd.	Greater visual cell damage than continuous light exposure	Organisciak, Jiang, Wang, Pickford, and Blanks (1989)
Rat R*attus norvegicus*	Lab	Five 1 min pulses every 2 hr using 2 standard 100 W incandescent lamps	Decreased peak night‐time serum melatonin concentrations. No effect on incidence or development of NMU‐induced mammary tumours	Travlos, Wilson, Murrell, Chignell, and Boorman (2001)
Syrian hamster *Mesocricetus auratus*	Lab	1 or 5 s pulse at 32,000 μW/cm^2^	Pineal melatonin production depressed	Reiter, Joshi, Heinzeller, and Nürnberger (1986)
Djungarian hamster *Phodopus sungorus*	Lab	1 min pulse at 40‐200 lx	Melatonin synthesis reduced during consecutive night	Lerchl (1995)
Social vole *Microtus socialis*	Lab	Three 15 min pulses at 450 lx	Resistance to cold markedly lowered	Zubidat, Ben Shlomo, and Haim (2007)
Nile grass rat *Arvicanthis niloticus*	Lab	1 hr pulse at 300 lx	Increased activity, & brain responses	Shuboni et al. (2015)
Mouse *Mus musculus*	Lab	1 hr pulse at 300 lx	Decreased activity, & brain responses	Shuboni et al. (2015)
Mouse *Mus musculus*	Lab	1 hr pulse at 100 lx	Decreased locomotor activity, increased anxiety & failure of memory performance, in proestrous females	Datta, Samanta, Sinha, and Chakrabarti (2016)

### 5.2 Developments

Globally, in 2012 there were an estimated 833 million passenger cars and 309 million commercial vehicles (OICA, 2014); these greatly outnumber streetlights, for example in the EU there are an estimated 287 million road vehicles and 60 million streetlights (The International Council on Clean Transportation, 2015; Van Tichelen et al., 2007). Clearly, particularly in developing economies, vehicle ownership is growing rapidly, and this trajectory is likely to continue. Since the growth in vehicle numbers is increasing at a greater rate than that of most countries' road networks, traffic density on the current roads will increase, which is likely to increase headlight pulse frequency by default. This said, the probable future trajectory of road transport and therefore volume is much debated. Whilst short‐term increases in car numbers are inevitable, the overall trends seem likely to be dependent on the type, rate and level of uptake of automated vehicles. Innovations could vary from advanced driver assist functions to full automation of personal cars and haulage vehicles. Full automation may perhaps appear in combination with a system where personal car ownership has all but ceased in urban areas, with rentable cars or taxis held in depots. In that case, the volume of traffic on roads could decrease, or become more evenly spread throughout the night‐time hours as passengers make long journeys in the (currently unpopular) small hours, by automated vehicle. Increases in night‐time traffic would obviously be a major concern for ecological impacts of headlights.

## 6 DISCUSSION

Widespread recognition of the, arguably pervasive, ecological impacts of artificial night‐time lighting has only emerged quite recently. Indeed, while spurred by key earlier contributions, the now rich literature of modeling, observational and experimental studies that documents these impacts has largely developed in the space of just the last decade. These insights have, however, focused almost exclusively on the consequences of emissions from static lighting sources. The argument that mobile sources, and especially those from road vehicle headlights, are both contributing substantially to overall levels of artificial night‐time lighting and to the ecological impacts seems compelling. Moreover, emissions from headlights give rise to particular concerns because of their intensity, predominantly horizontal and long trajectory, prevailing broad “white” spectrum, and the pulsed nature of the illuminance of habitat and organisms that they cause.

This said, it will be important to determine the details of the actual ecological impacts of emissions from vehicle headlights. In particular, it would be helpful to conduct field and mesocosm experiments with suitable study systems (e.g. see Bennie et al., 2015; Sanders et al., 2015), to measure the effects on individual organisms, populations and communities of pulsed lighting of different intensity, frequency and spectrum. Perhaps more so than with static lighting, a key challenge will be to determine the relative importance of, and interactions between, impacts of emissions from vehicle headlights and other ecological impacts of vehicles, including from traffic noise, exhaust emissions and animal roadkill. Disentangling these impacts may be difficult because the magnitudes of all will inevitably tend to be associated with traffic volumes. It would also be helpful to explore the impacts separately and in combination of emissions from streetlights and vehicle headlights, so as to unpick the likely consequences of night‐time vehicle use on roads with and without street lighting. Looking yet more broadly, one might embed such studies within a consideration of the overall ecological effect of roads, including on habitat availability and connectivity.

The practical challenges of reducing the ecological impacts of emissions from road vehicle headlights are perhaps greater than those associated with emissions from streetlights. First, the use of headlights is intimately associated with the night‐time visual needs of drivers and the avoidance of risks and discomfort of other road users. By contrast, streetlights serve a wide range of purposes, including safety, security, social benefit, and aesthetics, although their general importance for some of these (including impacts on levels of vehicle accidents and crime) is hotly disputed (Gaston, Gaston, Bennie, & Hopkins, 2015). Second, recent developments in headlight technology have not been strongly driven by concerns to further reduce energy demands (albeit there are clearly limits to what can be supplied) and carbon dioxide emissions, or further prolonging the life span of lamps. These factors have, however, been critical considerations in the development of street lighting schemes, particularly at a time when public finances are widely under great pressure following the global financial crisis (Gaston, 2013). Third, headlight technology has predominantly been focused on broad “white” spectrum lamps for a long time, on grounds of safety, and there seems little likelihood of changing this. By contrast, different parts of the world have employed different streetlight technologies, with different spectral characteristics, and the rapidity and extent, benefits and costs, of a switch to broad “white” spectrum lamps is a topic of much debate.

This is not to say that headlight systems could not be redesigned so as to better limit light emissions into places and in forms (e.g. intensities, spectra) that they are not needed. Recognition and understanding of the environmental consequences of these emissions is obviously a key to pressure for such changes. Substantial reduction in these environmental impacts will also require accompanying landscape or habitat changes that reduce the spread and influence of the light emissions from headlights, and the incorporation of such concerns into the planning and design of new roads.

## AUTHORS' CONTRIBUTIONS

K.J.G. conceived the idea; L.A.H. collected and analysed the data; K.J.G. led the writing of the manuscript. Both authors contributed critically to the drafts and gave final approval for publication.

## DATA ACCESSIBILITY

Data available from the Dryad Digital Repository https://doi.org/10.5061/dryad.q78sb73 (Gaston & Holt, 2018).
